# Nonequilibrium Colloids: Temperature-Induced Bouquet Formation of Flower-like Micelles as a Time-Domain-Shifting Macromolecular Heat Alert

**DOI:** 10.1021/acsami.3c09590

**Published:** 2023-09-07

**Authors:** Quirin Prasser, Thomas Fuhs, Bernhard Torger, Richard Neubert, Erica Brendler, Carla Vogt, Florian Mertens, Felix A. Plamper

**Affiliations:** † Institute of Physical Chemistry, TU Bergakademie Freiberg, Leipziger Straße 29, Freiberg 09599, Germany; ‡ Institute of Analytical Chemistry, TU Bergakademie Freiberg, Leipziger Straße 29, Freiberg 09599, Germany; § Center for Efficient High Temperature Processes and Materials Conversion ZeHS, TU Bergakademie Freiberg, Winklerstraße 5, Freiberg 09599, Germany; ∥ Freiberg Center for Water Research ZeWaF, TU Bergakademie Freiberg, Winklerstraße 5, Freiberg 09599, Germany

**Keywords:** aggregation, block copolymers, colloids, micelles, self-assembly

## Abstract

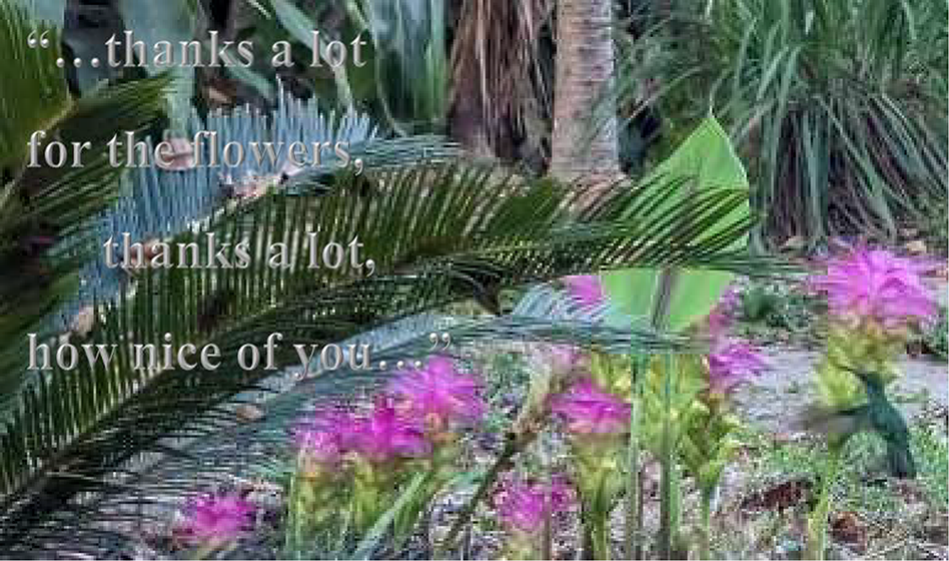

Climate change requires enhanced autonomous temperature monitoring during logistics/transport. A cheap approach comprises the use of temperature-sensitive copolymers that undergo temperature-induced irreversible coagulation. The synthesis/characterization of pentablock copolymers (PBCP) starting from poloxamer PEO_130_-*b*-PPO_44_-*b*-PEO_130_ (poly(ethylene oxide)_130_-*b*-poly(propylene oxide)_44_-*b*-poly(ethylene oxide)_130_) and adding two terminal *q*PDMAEMA_85_ (quaternized poly[(2-dimethylamino)ethyl methacrylate]_85_) blocks is presented. Mixing of PBCP solutions with hexacyanoferrate(III)/ferricyanide solutions leads to a reduction of the decane/water interfacial tension accompanied by a co/self-assembly toward flower-like micelles in cold water because of the formation of an insoluble/hydrophobic *q*PDMAEMA/ferricyanide complex. In cold water, the PEO/PPO blocks provide colloidal stability over months. In hot water, the temperature-responsive PPO block is dehydrated, leading to a pronounced temperature dependence of the oil–water interfacial tension. In solution, the sticky PPO segments exposed at the micellar corona cause a colloidal clustering above a certain threshold temperature, which follows Smoluchowski-type kinetics. This coagulation remains for months even after cooling, indicating the presence of a kinetically trapped nonequilibrium state for at least one of the observed micellar structures. Therefore, the system memorizes a previous suffering of heat. This phenomenon is linked to an exchange of *q*PDMAEMA-blocks bridging the micellar cores after PPO-induced clustering. The addition of ferrous ions hampers the exchange, leading to the reversible coagulation of Prussian blue loaded micelles. Hence, the Fe^2+^ addition causes a shift from history monitoring to the sensing of the present temperature. Presumably, the system can be adapted for different temperatures in order to monitor transport and storage in a simple way. Hence, these polymeric “flowers” could contribute to preventing waste and sustaining the quality of goods (e.g., food) by temperature-induced bouquet formation, where an irreversible exchange of “tentacles” between the flowers stabilizes the bouquet at other temperatures as well.

## 1 Introduction

Despite current issues concerning recycling and degradability, polymers have helped to increase the standard of living.^1^ In modern polymer research, stimuli-responsive, smart polymers react to environmental changes. A variety of triggers, like pH,^2^ light,^3^ ionic strength,^4^ or electricity,^5^ can cause precipitation,^6^ co/self-assembly,^7^ gel formation,^8^ etc. Temperature as a trigger is well investigated with poly(*N*-isopropylacrylamide) (PNIPAM) as an example.^9^ It can turn insoluble above a well-defined temperature called the lower critical solution temperature (LCST), which is the minimum temperature of a two-phase region in the phase diagram.^10,11^ Similar to poly(propylene oxide) (PPO), which reveals a variable LCST dependent on molar mass (oligomeric PPO_7_ has an LCST around 55 °C, PPO_18_ around 1 °C and the LCST of PPO_∞_with infinite degree of polymerization is estimated being close to −53 °C),^12−14^ the phase transition is fully reversible.^11^ In contrast, structural changes caused by a trigger can be irreversible in certain nonequilibrium systems^15−17^ (especially in nondissipative cases^18,19^): Metastable micellar structures exist in a local minimum of the free-energy landscape, thus triggers like temporary temperature changes^20^ can alter the pathway/kinetics toward the global minimum.^21^ Hence, irreversible morphological transitions can cause macroscopic changes like gelation.^20^ Though viscosity is surely a suitable readout to detect an intermediate application of a relevant trigger (like heat) in the history of a sample, an optical readout would be desirable, too. Therefore, this paper deals with the temperature-induced transition of a pentablock terpolymer qPDMAEMA_85_-*b*-PEO_130_-*b*-PPO_45_-*b*-PEO_130_-*b*-*q*PDMAEMA_85_ (*q*PDMAEMA: *quaternized* poly[(2-dimethylamino)ethyl methacrylate]; PEO: poly(ethylene oxide)) in the presence of ferricyanide ions (K_3_[Fe(CN)_6_]). Similar structures^22−24^ and the resulting flower-like micelles^25−27^ have been studied before, though kinetically trapped structures and transition between these structures at otherwise constant conditions were not in the main focus of these studies. The background of the current study is given by previously investigated PEO-*b*-*q*PDMAEMA and related polymers being unimolecularly dissolved in the absence of ferricyanide. Addition of multivalent counterions^28^ leads to co/self-assembly toward an optically transparent micellar dispersion being stabilized by solvated PEO.^5,29^ The introduction of PPO derived from commercial poloxamers enables a controlled coagulation of the PBCP above a threshold temperature,^30^ leading finally to turbid samples even at temperatures back below the threshold. Hence, the system behaves as a sensor with a memory effect, indicating whether the threshold was surpassed previously. Other systems can be used as polymeric “thermometers”, like l-proline-based polymers or PNIPAM-*g*-methylcellulose hydrogels showing a temperature hysteresis caused by hydrophobic interactions persisting in a certain interval below LCST.^31,32^ Poly(ferrocenylsilane)-based ionic liquids can show an LCST-like transition with hysteresis, where the transition is influenced by the redox state of the ferrocene.^33^ Another possibility are PNIPAM-based polymers with naphthalene moieties, where collapse retardation due to host–guest complexation leads to a memory window.^34,35^ Triblock copolymers exhibit a viscosity increase in the heat because of a transient network of flower-like micelles, where the exchange of hydrophobic blocks is impeded, preventing full reversibility.^36^ In contrast to these systems, our PBCP system performs an irreversible coagulation, reminiscent of the irreversible denaturing of proteins (as seen for e.g. egg-white). Compared to permanently hydrophobic end blocks, the preparation of the initial micellar dispersion is easily achieved by simple mixing of the components in cold water (without the need for a gradual change of solvent selectivity). The irreversibility of the transition can be switched off by the addition of ferrous ions, leading to colloidal Prussian blue hybrids,^8,37−40^ similar to the entrapment of perovskites in block copolymers.^41^ These effects might represent a quick, adaptable, and cheap method to monitor temperature for storage and transport purposes (the ingredients water, poloxamer, ferricyanide, and DMAEMA are rather inexpensive). Our method fits into the line of recent reports on sensors for temperature^42^ or food freshness.^43,44^

## 2 Experimental Section

### Materials

2-(Dimethylamino)ethyl methacrylate (DMAEMA), copper(I) bromide, Pluronic F108 (*M*_n_ 14 600 g mol^–1^), chloroform-d, potassium hexacyanoferrate(III), 4-(dimethylamino)pyridine (DMAP), methyl iodide, 1,1,4,7,10,10-hexamethyltriethylenetetraamine (HMTETA), iron(II) chloride tetrahydrate (>95%), and *n*-decane were purchased with the highest purity available from Sigma-Aldrich (Darmstadt, Germany). Copper(II) bromide and anisole were purchased from Alfa Aesar (Kandel, Germany). 2-Bromoisobutyryl bromide (97%) and basic aluminum oxide were purchased from Acros Organics. Tetrahydrofuran (THF), dichloromethane (DCM), and chloroform were purchased from Fisher Scientific (Schwerte, Germany). Triethylamine was purchased from Fluka (Munich, Germany). Silica gel 60 Å was purchased from Carl Roth (Karlsruhe, Germany); dioxane from Grüssing (Filsum, Germany); deuterium oxide from abcr (Karlsruhe, Germany); diethyl ether from Honeywell (Seelze, Germany); and potassium chloride from Chemsolute (Renningen, Germany). Dialysis membranes (regenerated cellulose Spectra/Por 7; MWCO = 1 kDa) were purchased from Carl Roth, Germany. A Merck Milli-Q IQ 7000 device delivered purified water for the preparation of samples.

The macroinitiator was synthesized from the commercial linear triblock copolymer Pluronic F108 (PEO_130_-*b*-PPO_45_-*b*-PEO_130_), which consists of a central poly(propylene oxide) block (PPO) connected with two hydroxyl-terminated poly(ethylene oxide) blocks (PEO), by esterification to yield a telechelic quaternary bromide for initiation during controlled radical polymerization. Pluronic F108 (*M*_n_ 14 600 g mol^–1^; 30 g), 4-(dimethylamino)pyridine (DMAP; 0.025 g; 0.0002 mol; 0.05 equiv), and triethylamine (4.6 g; 0.045 mol; 11 equiv) were dissolved in DCM (100 mL). After purging with N_2_ for 30 min, 2-bromoisobutyryl bromide (BiBB, 3.78 g; 0.016 mol; 4 equiv) was added dropwise while being stirred and cooled in ice. When the solution was at room temperature, another amount of 2-bromoisobutyryl bromide (1.89 g; 0.008 mol; 2 equiv) was added at 50 °C and stirred for 5 h. The resulting solution was diluted by 50 mL of DCM, filtered through a silica column, precipitated in diethyl ether three times, and freeze-dried from a dioxane solution for 2 d resulting in a white powder (Yield: 22.4 g; 75%).

The resulting macroinitiator was used for the polymerization, leading to a pentablock copolymer. Here, anisole (20 mL), DMAEMA (3.2 g; 0.02 mol; 215 equiv–with respect to the macroinitiating groups; both filtered over basic aluminum oxide), the macroinitiator (0.7 g; 5 × 10^–5^ mol), CuBr (0.009 g; 6 × 10^–5^ mol; 0.6 equiv), and CuBr_2_ (0.001 g; 5 × 10^–6^ mol; 0.05 equiv) were mixed and purged by N_2_ for 60 min. At 80 °C, HMTETA (1.5 equiv) was added, and the solution was stirred for 3 h. Prior to quenching with CHCl_3_, an NMR sample was taken in CDCl_3_ for conversion determination (40%, pointing to a theoretical number-average degree of a PDMAEMA block close to 85). The product solution was filtered over silica gel to remove the copper species, and the resulting solution was dialyzed against dioxane and freeze-dried for 2 days (Yield: 0.68 g, 33%).

Finally, the PDMAEMA block was quaternized according to known procedures. 0.6 g of the pentablock (0.0025 mol amino function) was dissolved in THF (50 mL). Methyl iodide (1.5 equiv) was added at 65 °C and the mixture stirred for 24 h. At room temperature, the solution was diluted with THF (20 mL), dialyzed against aqueous KCl solution (1 mol L^–1^) and pure water, and freeze-dried for 2 d (Yield: 0.42 g; 60%).

### ^1^H NMR Spectroscopy

^1^H NMR spectra were recorded from all products and interim products using either CDCl_3_ or D_2_O as a solvent with a BRUKER Nanobay 400 MHz spectrometer (Bruker, Ettlingen, Germany). The chemical shifts are indicated in parts per million in respect to the tetramethylsilane (TMS) standard. The solvent signals are used as reference.

### Emergent Drop

Emerging drop measurements at the water/*n*-decane interface were performed to determine the interfacial tension using a DSA 100 drop shape analyzer (Krüss, Hamburg, Germany). The DSA works by evaluating the shape of an emerging oil droplet in the aqueous solution. Three solutions containing always KCl (0.1 mol L^–1^) were examined: Pluronic F108 (1 g L^–1^) and PBCP (1 g L^–1^) were mixed with K_3_[Fe(CN)_6_] (0.0025 mol L^–1^) and without it. For preparation of the ferricyanide-containing solutions, KCl solutions containing a double concentration of PBCP and K_3_[Fe(CN)_6_] were prepared separately before mixing equal amounts of the two solutions. The temperature of the aqueous solution was varied from 10 °C at minimum to 40 °C at maximum, if the drop shape analysis was possible (precipitation of the PBCP/ferricyanide made the measurements impossible). All measuring points were measured three times while the outer solution was gently stirred. Because of the long equilibration times, the measuring time was 4000 s after the generation of the drop, where the interfacial tension curve has flattened.

### Dynamic Light Scattering

Dynamic light scattering measurements were performed with an ALV/CGS-3 Goniometer system (Langen, Germany) equipped with a He·Ne laser (32 mW, 632.8 nm), two avalanche photo diodes, an external programmable thermostat (Julabo F32), and an index-match bath filled with toluene. Angle- and temperature-dependent measurements were performed in a pseudocross-correlation mode, varying the scattering angle from 30° and 150° in 10° steps, 3-fold, with a duration of 30 s for each run. Since all samples were monomodal, their decay rates obtained as the first cumulant of the second-order cumulant fit were plotted against the squared scattering vector *q*^2^. The data were fitted by a linear regression, where the diffusion coefficient was extracted as the slope, and *R*_H_ was calculated with the Stokes–Einstein equation.

### Small Angle X-ray Scattering (SAXS)

SAXS experiments of the PBCP system were performed at a SAXSpoint 5.0 beamline (Anton Paar, Graz, Austria) equipped with a Primux 100 microfocus X-ray source (Cu Kα radiation; λ = 1.54 Å), ASTIX 2D multilayer X-ray optics, and a 2D EIGER2 R 1 M hybrid photon counting detector shielded with a mylar film (Dectris, Baden, Switzerland). The samples were measured in a temperature controlled TCstage 600 (Anton Paar, Graz, Austria) equipped with a rotor cell at 25 and 35 °C with a sample-to-detector distance of 1627 mm using plastic capillaries under rotation. All data were fitted by the software SasView 5.0.5.^45^

### Drop Casting/Atomic Force Microscopy

Samples of the solution for atomic force microscopy (AFM) were prepared by drop casting. A droplet of the solution of interest was placed on a fresh silicon wafer for 1 min. The solution was carefully absorbed by paper without touching the surface of the wafer in order to prevent the formation of KCl crystals at the surface. After the preparation, the samples dried for at least 3 h. AFM images of the coated substrates were taken via a Park Systems NX10 (Suwon, Korea) in noncontact mode using MikroMasch 160AC-NA (Sofia, Bulgaria) cantilevers. The samples were measured with resolution of 256 × 256 pixels, on an area of either 10 × 10 μm^2^ or 2 × 2 μm^2^, at different positions to ensure reliability of obtained data. Images were processed by Gwyddion software.^46^

### Size-Exclusion Chromatography (SEC)

SEC measurements in dimethylacetamide (DMAc) were conducted on a PSS SECcurity^2^ system consisting of a PSS SECurity two-channel-inline-degasser, a PSS SECurity^2^ TCC6500 column oven (50 °C), a PSS GRAM column set (8 × 50 mm, 10 μm precolumn, 8× 300 mm, 10 μm analytical linear columns), an PSS SECcurity^2^ isocratic pump, an PSS SECurity^2^ automatic vial sampler, and an PSS SECcurity^2^ refractive index detector (35 °C). High-performance liquid-chromatography-grade DMAc is used as an eluent at a flow rate of 1 mL min^–1^. PSS ReadyCal-Kit PEO/PEG with *M*_p_ 238–966 000 Da were used as calibrants. All samples were prepared in concentrations of ∼1 mg mL^–1^ (in DMAc) and passed over 0.22 μm poly(tetrafluoroethylene) membrane filters. Molecular weight and dispersity analysis were evaluated with PSS WinGPC UniChrom software (version 8.2).

## 3 Results and Discussion

### Synthesis

**Figure 1:**
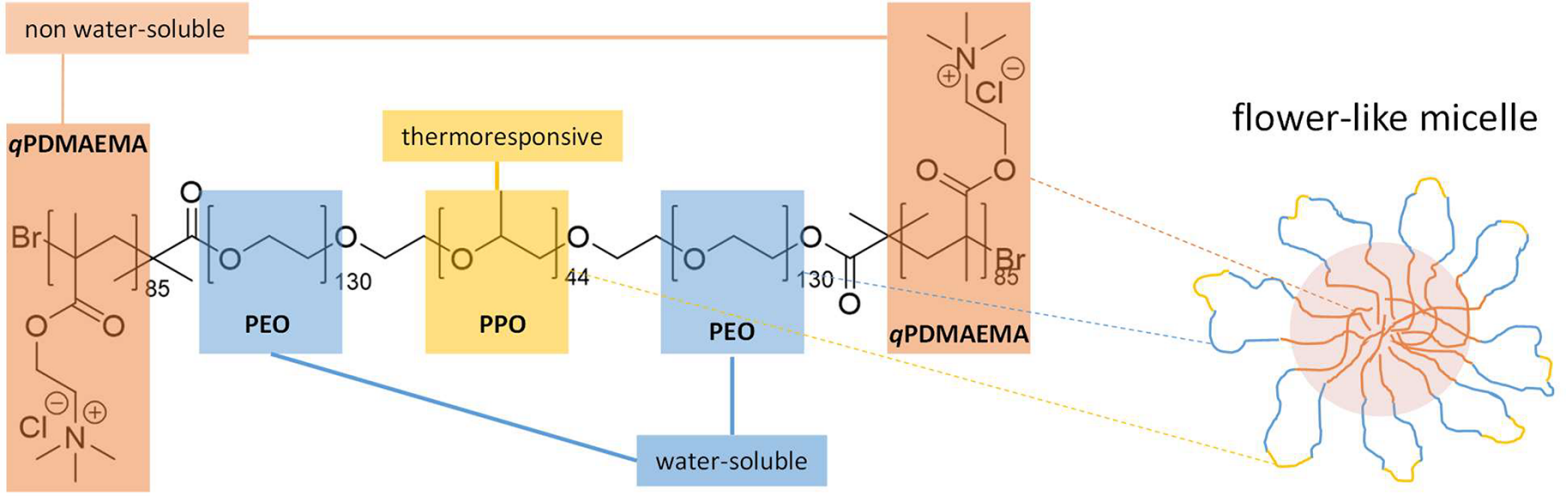
Molecular structure of the synthesized PBCP and schematic representation of the expected micellar structure (*q*PDMAEMA is insoluble in the presence of ferricyanide).

A pentablock copolymer (PBCP) was synthesized by atom transfer radical polymerization from the commercial poloxamer Pluronics F108 resulting in *q*PDMAEMA_85_-*b*-PEO_130_-*b*-PPO_45_-*b*-PEO_130_-*b*-*q*PDMAEMA_85_ (Figure 1).^47^ The synthesis followed the instruction of related diblock copolymer syntheses.^5,48,49^ The synthesis consisted of three steps (Figure S1), namely the conversion of the initial triblock copolymer to a 2-fold macroinitiator, the controlled polymerization, and the methylation of the amino group of the terminal PDMAEMA blocks. The nonquaternized intermediates were characterized by ^1^H NMR spectroscopy in CDCl_3_ and SEC to verify the success of the polymerization and determine the number-average degree of polymerization of the *q*PDMAEMA blocks *n* = 85 (SI Figure S2/S3). The quaternized product^49^ with a total molecular weight *M*_n_ = 49.8 kg mol^–1^ (including chloride counterions) was characterized by ^1^H NMR spectroscopy in D_2_O (see SI Figure S2). Further details of synthesis and molecular characterization are given in the Supporting Information (SI).

### Interfacial Behavior

**Figure 2:**
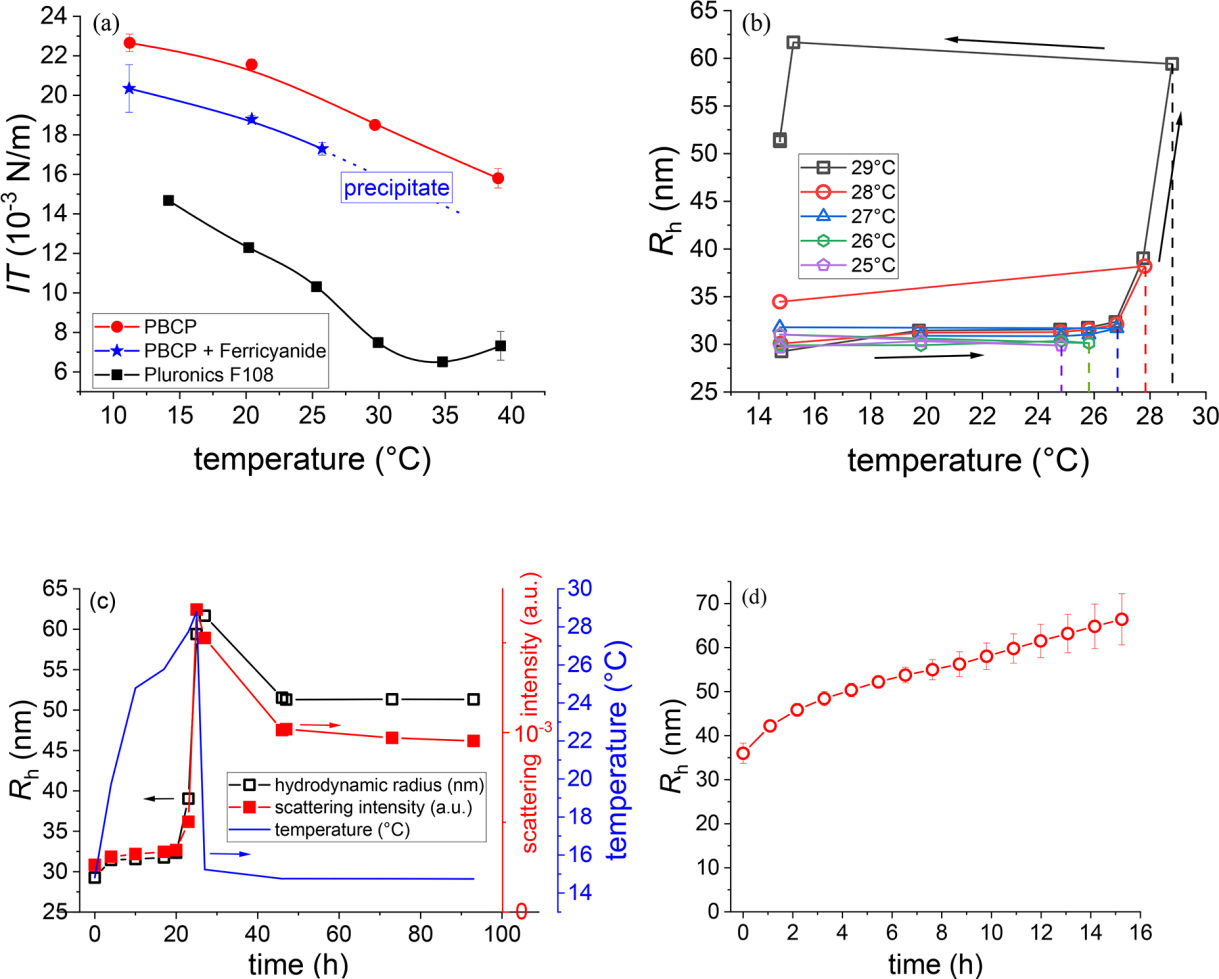
Interfacial and solution behavior of Pluronic F108 (0.5 g L^–1^), PBCP (1 g L^–1^), and K_3_[Fe(CN)_6_] (0.0025 mol L^–1^) in aqueous KCl (0.1 mol L^–1^). (a) Temperature dependent interfacial tension of the water/*n*-decane interface of the triblock copolymer Pluronic F108 (black), PBCP in the presence (blue; flower micelles) and absence (red) of K_3_[Fe(CN)_6_]. (b) Hydrodynamic radii (*R*_H_) of the aqueous PBCP/K_3_[Fe(CN)_6_]/KCl solutions during the heating process: all samples were prepared below 15 °C and heated to the target temperature separately (heating rate ∼2 K/h); equilibration time was 5 min, and the measurement took between 15 and 25 min to measure scattering angles between 30 and 150° in 10° steps for 10 s. (c) Time-dependent development of the temperature, *R*_H_ and the scattering intensity at 90° of PBCP/K_3_[Fe(CN)_6_]/KCl solution heated to 29 °C. (d) Time-dependent increase of the average *R*_H_ at 27.5 °C; 4 curves of a cooled PBCP/K_3_[Fe(CN)_6_]/KCl stock solution were analyzed until multiple scattering influences the measurement (lines are a guide to the eye).

The interfacial activity and micellization behavior of poloxamers is well studied.^50,51^ Also we investigated the interfacial properties of polymers before.^5,8,48,52^ It is known that the adsorption behavior of poloxamers follows the concept of a hydrophilic–lipophilic balance (HLB). Hence, the water/air surface activity increases with higher amount of hydrophobic interactions.^53^ In Figure 2a, temperature-dependent values of the water/*n*-decane interfacial tension are plotted in the presence of Pluronic F108 and the pentablock copolymer *q*PDMAEMA_85_-*b*-PEO_130_-*b*-PPO_45_-*b*-PEO_130_-*b*-*q*PDMAEMA_85_ (PBCP) with and without potassium ferricyanide (as determined by drop shape analysis). The interfacial tension (*IT*) of aqueous solutions of Pluronic F108 showed a significant reduction in contrast to the pure interface (*IT* = 52 × 10^–3^ N m^–1^, 25 °C)^54^ or the PEO_110_ decorated interface (*IT* = 31 × 10^–3^ N m^–1^, 25 °C).^8^ The polyelectrolytic *q*PDMAEMA blocks of PBCP are well water-soluble in absence of multivalent counterions.^5,55^ Consequently at 25 °C, the increase of interfacial tension from ∼ 10 × 10^–3^ N m^–1^ for Pluronic F108 to ∼20 × 10^–3^ N m^–1^ for PBCP solutions is explained by the shift in HLB toward hydrophilic interactions. This is in line with the upper limit of a *q*PDMAEMA_80_-decorated decane/water interface, which exhibits an *IT* of 36 × 10^–3^ N m^–1^ (25 °C).^5^*q*PDMAEMA blocks are known to change their solubility in the presence of certain multivalent counterions as seen for the formation of micelles in the presence of ferricyanide.^3,29^ This is attributed to a complexation of certain multivalent counterions, thus the *q*PDMAEMA is hydrophobized by ferricyanide as shown for other interfacially active derivatives of PDMAEMA at interfaces.^5^ In the current case, the addition of ferricyanide leads to a reduction of the interfacial tension by 2–3 × 10^–3^ N m^–1^. These changes lie in the range observed for ferricyanide additions to a more amphiphilic, polycationic block copolymer system.^5^ Noteworthy, the *IT*-values are either unreliable or simply not measurable any more beyond a temperature threshold of ∼ 28 °C in the presence of ferricyanide ions, since the pentablock copolymer precipitates rapidly (Figure 2a). This threshold-temperature is consistent with the critical micelle temperature of aqueous Pluronic F108 solutions, since micelles are detected above the cloud point of the PPO block.^56^ Hence, solutions of PBCP and potassium ferricyanide reveal a critical precipitation temperature, in contrast to solutions of the pure poloxamers.

### Solution Behavior

The difficulties during drop shape analysis (see Figure 2a), which remained even after cooling, moved our focus to the solution properties of the micellar dispersions. Hence, dynamic light scattering (DLS) measurements were performed while stepwise increasing the heating target temperature between 25 and 29 °C and subsequent cooling to 15 °C. This allows the determination of the temperature-dependent development of aggregates in the PBCP solution (1 g L^–1^, 0.0025 mol L^–1^ K_3_[Fe(CN)_6_], 0.1 mol L^–1^ KCl; see Figure 2b). In cold water, the PEO/PPO blocks are expected to be hydrophilic, while the terminal *q*PDMAEMA/ferricyanide blocks lead to flower-like micelles, where PEO-*b*-PPO-*b*-PEO loops/petals surround the insoluble core. The hydrodynamic radius (*R*_H_) of the micellar aggregates is constant at temperatures between 15 and 27 °C. Beyond this temperature range, the aggregates grow rapidly. After cooling, the size of the aggregates decreases slightly but remains essentially constant above the initial size. Further samples heated to temperatures beyond 27 °C turned turbid with time, thus DLS can be questionable as multiple scattering distorts the results. In Figure 2c, the development of temperature, *R*_H_, and scattering intensity with the highest evaluable target temperature is plotted. After an initial slight decrease of *R*_H_ after cooling back to 15 °C, the aggregates have no tendency to shrink further. The initial decrease could be due to a limited reversal of the thermo-induced coagulation. The similar scattering intensity trend is in line with some reversal, as newly aggregated material is not yet strongly bound. Sedimentation seems not play a major role in this temperature/time range, though we cannot exclude sedimenting particles because of a broad size distribution. The growth accelerates at high temperatures. This effect leads to a rapid coagulation/precipitation aggravating DLS analysis at temperatures beyond 29 °C. Between these temperatures, the change of *R*_H_ is slow enough to record the evolution (Figure 2d; for data treatment, see SI Figure S4). The first measurement shows a *R*_H_ around 35 nm followed by a constant increase before the solution turned turbid after 14–16 h.

To examine the stability of the solution, a sample was heated to 35 °C for 10 min, leading to a dense coagulation. The sample was not measurable by DLS even after more than three months of storage at 4 °C, where the sedimented precipitate kept the yellowish color of ferricyanide. A nonheated, yellowish sample stored for three months at 4 °C exhibits an *R*_H_ of 34 nm and transparency, envisioning applications for months.

**Figure 3:**
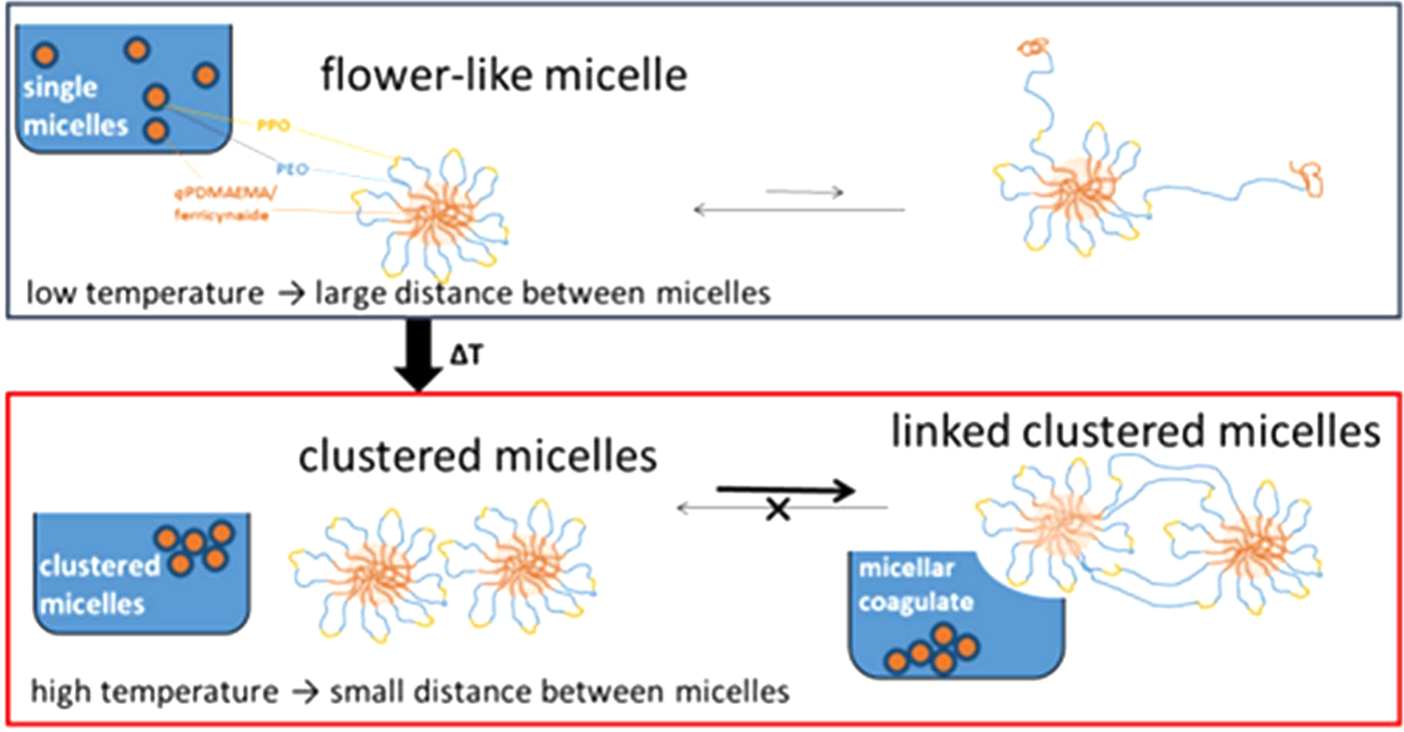
Schematic illustration of a mechanism of temperature-induced coagulation. In cold water, a dynamic equilibrium of spatially separated flower-like micelles exists. In hot water, the PPO blocks collapse leading to clustering, where the exchange of hydrophobic blocks bridges different micellar cores.

An explanation attempt is given with the help of an exchange mechanism of insoluble *q*PDMAEMA/ferricyanide blocks between the micelles, where the micellar core is surrounded by loops of PEO-*b*-PPO-*b*-PEO, being solvated at low temperatures (Figure 3). At low concentrations, the distance between the micelles is large. In hot water, the PPO at the rim of the micelles collapse resulting in sticky “tentacles”, which lead to an accumulation and eventually to a precipitation. Cooling would redisperse the micelles. The irreversibility can be explained by the dynamics of the system. Similar to low molecular weight surfactants, hydrophobic groups are in equilibrium between a solubilized and a micellized state. In case of PBCP, the solubilization of one block is more likely than of a whole unimer. In cold water, this equilibrium does not affect the colloidal stability since a *q*PDMAEMA/ferricyanide block will not interact with other micelles, but steric repulsion keeps the micelles apart. In the clustered regime beyond the LCST of PPO, a hydrophobic *q*PDMAEMA/ferricyanide block can be embedded in another core, forming a bridge. The transition is sharp (Figure 2), which is explained by the defined cloud point of PPO. Cooling below the LCST would stop the growth of the clusters but not redisperse them because of the bridging.

An alternative explanation attempt based on thermo-induced (and irreversible) complexation between PPO and *q*PDMAEMA^57^ is tested by NMR experiments (see SI for a more detailed discussion; Figure S6). This complexation between PPO and PDMAEMA was observed before for miktoarm star polymers in solution and for block copolymers at interfaces.^57^ In the current case, the complexation would lead to a change in the HLB and hence to morphological changes of the core and therefore of the whole self-assembly toward larger wormlike or vesicular micelles, which could eventually cause turbidity. However, NMR shows no indication of any dominant interaction and complexation between PPO and *q*PDMAEMA (Figure S6).

### Structural Elucidation

**Figure 4:**
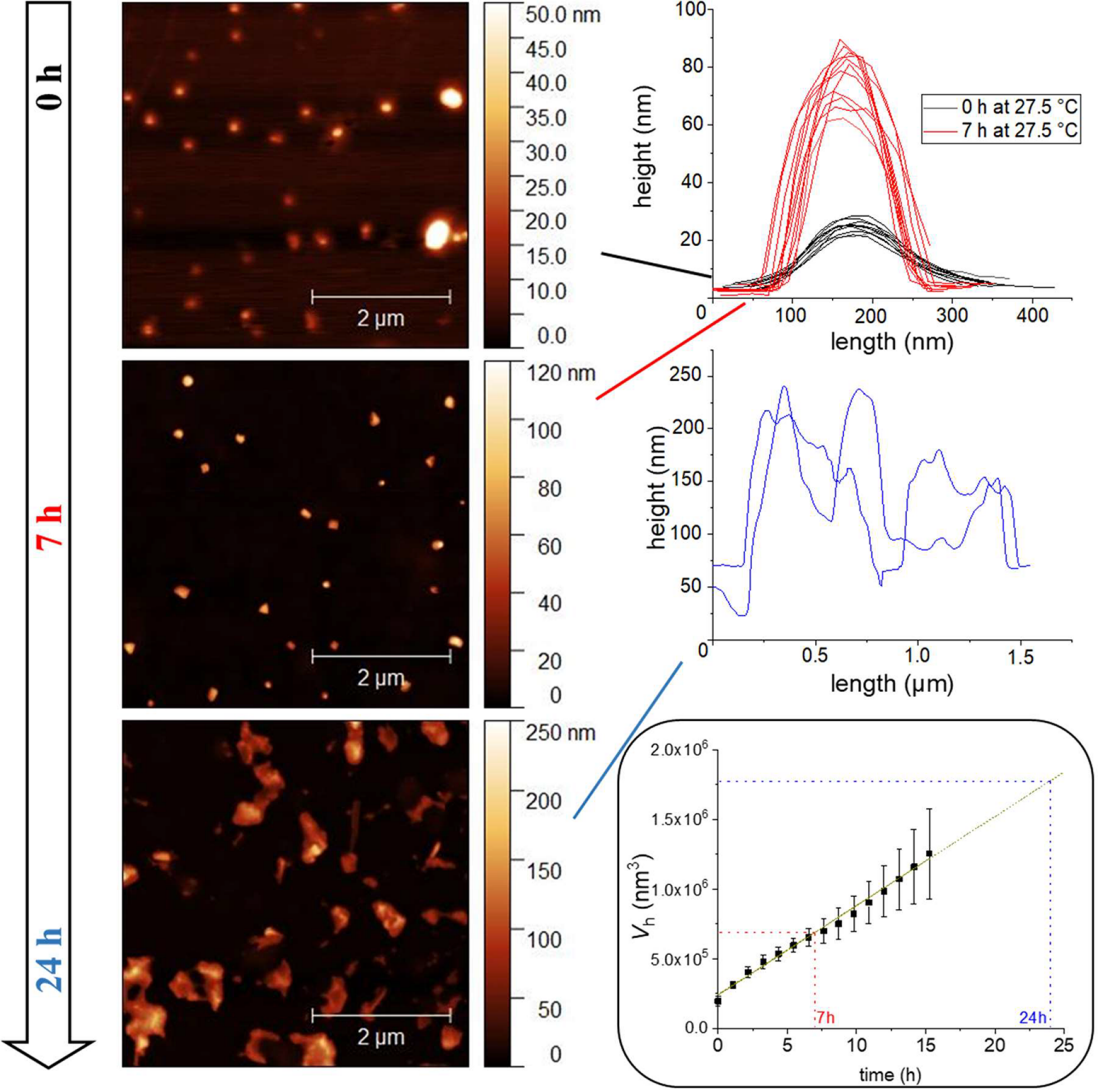
AFM images of a solution of PBCP (1 g L^–1^, 0.0025 mol L^–1^ K_3_[Fe(CN)_6_], 0.1 mol L^–1^ KCl) transferred to a silicon wafer by drop casting after 0 h, 7 h, 24 h at 27.5 °C (left) and a selection of height profiles (right); time-dependent development of hydrodynamic volume  as calculated from Figure 2d including marks for the deposition times (after 16 h, the values were linearly extrapolated, as predicted by Smoluchwoski;^58^ right, bottom).

To gain more structural insight, Atomic Force Microscopy (AFM) was performed after drop casting the PBCP/K_3_(Fe(CN)_6_)/KCl dispersion onto silicon wafers (Figure 4 and Figure S5). For investigating the structures by AFM after transfer onto a solid substrate, the solution was prepared at temperatures below 15 °C and heated to 27.5 °C for 24 h. Transferred samples were taken directly after preparation and heating, after 7 h, and after 24 h. At the last sample, the solution was turbid. In Figure 4, the evolution of structures is depicted as images and height profiles (for selection of measured height profiles, see SI, Figure S5). Initially, the micellar volume is in the same range as the micelles detected by DLS. Within the first 7 h, flattened spheroidal particles are discernible, which significantly grew over time. Despite a deviation in the maximum height of the particles detected on one initial sample, the primary particles (height 20–30 nm) are clearly distinguishable from the particles after 7 h (height 60–85 nm). The volume per micellar aggregate has increased by a factor ∼3, indicating that the particles after 7 h consist of approximately 3 original micelles. At the same time, they seem to be compacted and less deformable, probably because of the additional physical cross-links between the PPO patches (and partly bridged *q*PDMAEMA/ferricyanide domains). This interaction leads probably also to a rather narrow distribution of sizes, which is not necessarily expected from classical Smoluchowski coagulation theory.^58,59^ In line with the theory, the average cluster size increases almost linearly with time, at least in the first 16 h of coagulation (the typical coagulation time τ for the observed retarded coagulation is in the range of 3.5 h at 27.5 °C). This was demonstrated by DLS (Figure 3), where the increase in size detected by AFM fits nicely to the 3-fold increase in hydrodynamic volume *V*_h_, as shown for 7 h. The continuous growth of the particles is consistent with the intermicellar aggregation caused by sticky PPO domains. At later stages, deviations from the linear growth of *V*_h_ are expected, as *V*_h_, which also includes nondrained solvent, is not directly correlated to the actual volume of the set of micelles being aggregated to form a larger cluster/fractal. Indeed, the shape of the particles appears different after 24 h, since they are not uniform but broadened over a large area, and they show several maxima. Note that the last sample was taken when the particles were not fully colloidally stable; thus, larger particles and less symmetric shapes with higher interfacial area are detectable. The various maxima of an aggregate after 24 h can be attributed to different micelles in one cluster, where several micelles are linked by polymer chains. Noteworthy, the aggregation at longer time scales seems to deviate from the classical Smoluchowski behavior, since the sizes detected by AFM after 24 h are considerably larger than the ones expected from the extrapolated initial kinetics. Here, not only the arising fractal structures but also an increasing contribution of sedimentation could change the kinetics. At temperatures beyond 29 °C, the coagulation shifts from retarded kinetics (with a potential barrier due to steric repulsion) to more rapid diffusion-limited kinetics, leading to a nearly instantaneous turbidity of the sample. Hence, prolonged exposure at moderately warm temperatures (slightly above threshold) has the same effect as a short extensive heat wave. This behavior is deemed desirable for the monitoring of heat-sensitive samples, where the damage depends on both the time and extent of the detrimental exposure.

**Figure 5:**
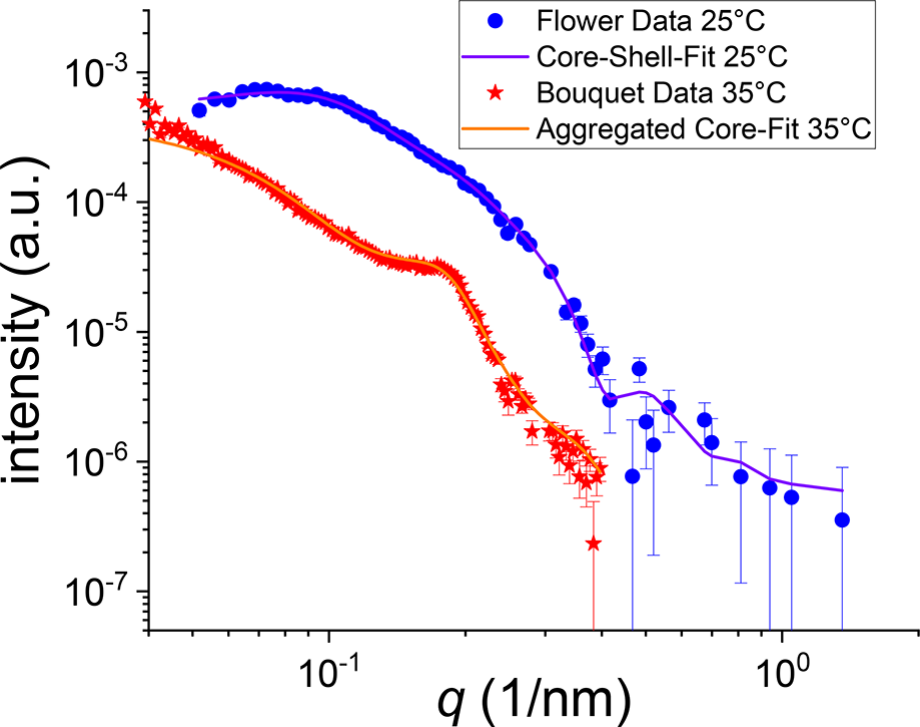
SAXS data (solvent subtracted) and fits of an aqueous solution containing PBCP (1 g/L), K_3_[Fe(CN)_6_] (0.0025 mol L^–1^), and KCl (0.1 mol L^–1^) at 25 and 35 °C. Curves were recorded from samples in a rotating capillary to enable measurements of a sedimenting suspension. For the measurements at 25 °C (blue circles; measurement time shortened to 30 min to guarantee constant temperature), a core–shell form factor with a sticky-hard-sphere structure factor was used (purple line). For measurements at 35 °C, the curve (red stars; measured for 5 h and normalized to 30 min measurement time) was fitted with a hard sphere form factor with a hard sphere structure factor (orange line).

To gain more insight into the colloidal structures, small angle X-ray scattering (SAXS)^60^ data was obtained prior to heating and after cooling again (Figure 5; for deeper discussion on the models including the fit results, see the Supporting Information).

The data before heating are in line with flower-like micellar structures, as the data can be modeled with a core–shell form factor model supplemented by a sticky-hard-sphere structure factor. Here, the thermal motion and the corona repulsion still prevail, and the particles do not aggregate permanently at low temperatures. At 25 °C, the sample shows a scattering behavior of a dispersion of core–shell^61^ particles having a core radius of 10 nm and a shell thickness of 16 nm. These values are in line with the contour lengths of the involved polymer blocks, which would constitute upper limits for the core radius and shell thickness. The total radius (26 nm) is slightly below that of DLS measurements, which also includes bound water molecules and dangling loops/chains not seen in SAXS.

After the sample was heated to 35 °C and held at that temperature for 20 min, the measurement was repeated, leading to a different scattering (bouquet). The curve showed two main differences. The decay was shifted to lower *q*-values indicating larger structures and a structure peak arose at *q* = 0.18 nm^–1^ (corresponding Bragg-distance: 35 nm). Please note that the precipitating dispersion was measured in a rotating cell, allowing also the investigation of sedimenting samples. Because of the limited *q*-range and large aggregate sizes, the overall morphology/dimension of the heated clusters/fractals cannot be resolved by SAXS, but information about the internal structure can be extracted. Here, the fit model used for the low temperature data (flowers) was adapted for the high temperature part (bouquet) by using a hard sphere form factor with hard-sphere structure factor after realizing that a core–shell fractal model^62^ did not describe the data successfully. The average core radius increased slightly to 13 nm compared with the 10 nm radius of the scattering-dominant cores before heating (which now includes also the scattering of the shell in the model used for the heated sample). In addition, the scattering behavior can only be fitted when assuming a greatly increased polydispersity of the core radius. This result is in line with some exchange of the insoluble chains and the possible formation of secondary cores, either made of *q*PDMAEMA/ferricyanide or collapsed PPO. This aggregation leads also to a smaller distance between the cores (Bragg-distance at 35 nm) compared to the double radii obtained by DLS or SAXS for single flower-like micelles. In summary, the SAXS measurements confirm the results of DLS and AFM experiments by indicating a cluster formation of core–shell particles above the threshold temperature of 27 °C.

### Prussian Blue Formation

**Figure 6:**
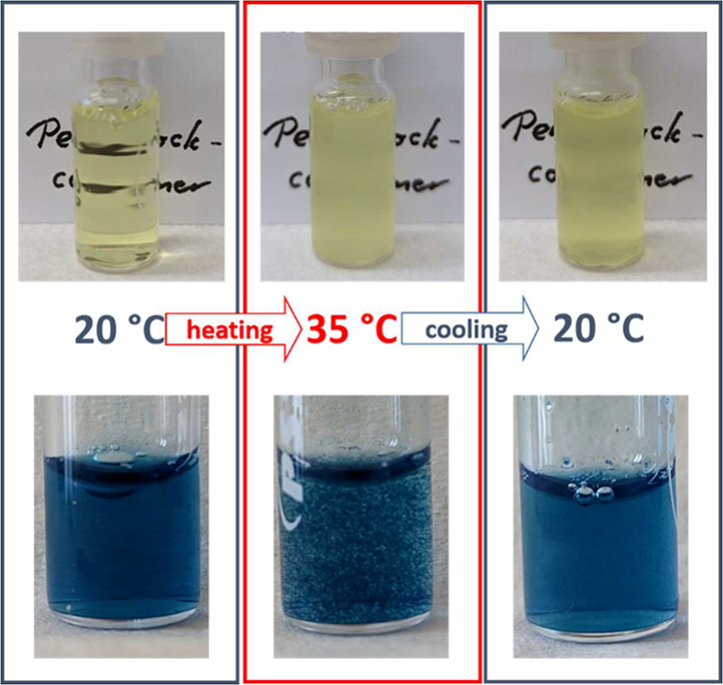
Pictures of PBCP solutions (1 g L^–1^, 0.0006 mol L^–1^ K_3_[Fe(CN)_6_], 0.1 mol L^–1^ KCl, yellow top), with iron(II) chloride (0.006 mol L^–1^) added to form PB (blue bottom); the solutions were prepared below 20 °C, heated for 5 min to 35 °C, and subsequently cooled to 20 °C.

To corroborate the proposed mechanism, the dynamics of the micellar core can be restricted by the formation of solid Prussian Blue (PB) nanoparticles.^8^ For this purpose, iron(II) chloride was added to the cooled solution, where coagulation had not yet occurred (Figure 6; for details, see the Supporting Information). The linkage between the resulting PB and the polymer is demonstrated by heating of the transparent, blue dispersion, which leads to a coagulation toward particles observable by the mere eye, similar to the solution without iron(II), which became turbid under the same conditions. Hence, the precipitation behavior during heating is not considerably changed because of the formation of PB, since it is caused by the collapse of the PPO chains at the rim of micelles. During cooling of the precipitated samples to 20 °C, the PB containing sample formed a transparent solution again in contrast to the sample without PB. This behavior is consistent with the previously explained hypothesis, since the mobility of the nonsoluble *q*PDMAEMA/ferricyanide blocks is sufficient enough for their exchange between micelles, which makes the precipitation irreversible. By embedding the chains at and/or in solid nanoparticles, the exchange is inhibited, and thus, the temperature-induced precipitation is reversed by cooling below the cloud point of the PPO block. At the same time, the colloidal stability of PB can now be reversibly adjusted by temperature.

### Possible Applications

Apparently, the system remembers “heat waves” via changes in its solution behavior. Hence, the system could be used as a temperature sensor with memory, since temperatures beyond the threshold can be detected afterward by mere eye sight. The dispersion would be suitable for controlling the cold chain of temperature-sensitive samples (e.g., medical samples, live tissue, or just food), since a small, transparent vial, which could be embedded in a tag or forgery-proof sticker, would document the temperature history without the need of additional sensors, power supplies, and devices for digital logging. The threshold depends on the LCST-type cloud point of the PPO block.^56^ Its transition temperature decreases with higher PPO chain lengths.^63^ Consequently, the use of poloxamers with different block ratios should lead to a variation of the threshold, providing adaptability to different application scenarios. Then, such temperature sensors can be beneficial for industry and logistics. Noteworthy, the addition of ferrous ions leads to a shift from the monitoring of the thermal past toward the monitoring of the current temperature state.

## 4 Conclusion

The synthesis of an *q*PDMAEMA-*b*-PEO-*b*-PPO-*b*-PEO-*b*-*q*PDMAEMA block copolymer revealed a versatile substance showing a variety of useful properties in suitable environments. The interfacial activity at a water/oil interface can be controlled by the addition of potassium ferricyanide or a variation of the temperature in a certain temperature range. Considering the bulk phase of a KCl solution containing the PBCP and potassium ferricyanide, the hydrodynamic radius of the resulting micelles remains constant at low temperatures. Above a threshold temperature of 27 °C, the aggregates grow with a temperature-dependent rate yielding larger aggregates and finally a precipitate, likely consisting of linked clusters of micelles. The transition from flower-like micelles toward insoluble micellar clusters is explained by a temperature-induced local accumulation due to the temperature-responsive PPO blocks enabling an exchange of hydrophobic *q*PDMAEMA/ferricyanide block between micellar cores. Hence, the micellar cores are bridged resulting in an irreversible clustering of the structures. This process was confirmed by AFM-images of the solution, drop casted on a silicon substrate. As long as the structures are small (unimers, dimers, trimers, etc.), spheroidal aggregates are found in solution, while the precipitate at prolonged times shows larger and irregular or even fractal structures from the association of several micelles. The exchange of hydrophobic blocks could be avoided by the formation of Prussian blue nanoparticles in the micellar cores restricting the dynamics of these blocks. By this way, a temperature induced precipitation of Prussian blue took place, but became reversible by cooling the system below the threshold temperature, which corroborates the hypothesis of a block-exchange within a micellar cluster. Since the system responds sensitively at temperatures above a certain value with the irreversible formation of aggregated structures, it can be considered as a temperature sensor indicating whether or not the threshold temperature has been exceeded in the past. Further research is needed to examine the versatility of such PBCPs, since a variation of the composition and the block lengths (especially the PPO block) might enable an adaption of the threshold temperature. Because of the sensitive and irreversible temperature response, PBCP/ferricyanide solution can be beneficial for industry and logistics as temperature sensors with a memory that is independent from the electricity supply.

## Supporting Information Available

A detailed description of the synthesis and characterization of the polymers including NMR and SEC; data averaging of the time-dependent DLS data, selection of the height profiles obtained by AFM, description of the SAXS modeling, discussion of an alternative mechanism of precipitation, formation of Prussian blue (PDF)
Several data files of plotted data (ZIP)
