# Supplementary material for: Nonequilibrium Colloids: Temperature-Induced Bouquet Formation of Flower-like Micelles as a Time-Domain-Shifting Macromolecular Heat Alert
Source: ACS Appl Mater Interfaces. 2023 Sep 7;15(50):57950–9. doi: 10.1021/acsami.3c09590 (PMC10739602; doi:10.1021/acsami.3c09590)
Supplement: Supplementary file 1 — am3c09590_si_001.pdf [file am3c09590_si_001.pdf]

Supporting Information to

Nonequilibrium Colloids: Temperature-Induced Bouquet Formation of Flower-like Micelles as a Time-Domain-Shifting Macromolecular Heat Alert

Quirin Prasser,[a] *Thomas Fuhs,*[a] *Bernhard Torger,*[a] *Richard Neubert,*[a] *Erica Brendler,*[b] *Carla Vogt,*[b] *Florian Mertens,*[a,c] *Felix A. Plamper**[a,c,d]

[a] Institute of Physical Chemistry, TU Bergakademie Freiberg, Leipziger Str. 29, 09599 Freiberg, Germany

E-mail: felix.plamper@chemie.tu-freiberg.de

[b] Institute of Analytical Chemistry, TU Bergakademie Freiberg, Leipziger Str. 29, 09599 Freiberg, Germany

[c] Center for Efficient High Temperature Processes and Materials Conversion ZeHS, TU Bergakademie Freiberg, Winklerstr. 5, 09599 Freiberg, Germany

[d] Freiberg Center for Water Research ZeWaF, TU Bergakademie Freiberg, Winklerstr. 5, 09599 Freiberg, Germany.

Synthesis: The synthesis of PDMAEMA-*b*-PEO-*b*-PPO-*b*-PEO-*b*-PDMAEMA pentablock copolymers was performed according to the instructions of Steinschulte et al.[1] for the synthesis of PEO-*b*-PDMAEMA diblock copolymers and Bütün et al.[2] for the selective quaternization of poly(dialkyl amino)ethyl methacrylate blocks (Figure S1).

The macroiniator was synthesized from hydroxy-terminated PEO_{130}-*b*-PPO_{44}-PEO_{130} (Pluronic F108 (apparent M_n=13 Kg·mol^{-1} obtained by SEC against PEO calibration; $Đ$=1.17) by help of 2-bromo isobutyryl bromide (BiBB), yielding 22.4 g of triblock copolymer with two initiating sites. The product was analyzed by ^{1}H-NMR spectroscopy and SEC.

An atom-transfer radical polymerization (ATRP) was performed to transform the macroinitiator into the pentablock terpolymer PDMAEMA-*b*-PEO-*b*-PPO-*b*-PEO-*b*-PDMAEMA. For this purpose, the monomer 2-dimethylaminoethyl methacrylate (DMAEMA) (215 equiv) reacted with the macroinitiator in anisole with help of the catalyst/activator CuBr and deactivator $CuBr_2$ at 80 °C after addition of 1,1,4,7,10,10-hexamethyltriethylenetetraamine (HMTETA) as ligand. After purification and freeze drying, the polymer (0.68 g; yield 33 %) was characterized by ^{1}H-NMR spectroscopy and SEC. According to ^{1}H-NMR of the non-purified product solution (comparison of the integrals of signals of PDMAEMA and monomeric DMAEMA), the conversion was 40 % (implying a theoretical PDMAEMA block degree of polymerization as 85), the corresponding number average degree of polymerization of each PDMAEMA block is 77 (determined by NMR endgroup analysis taking the PEO signal as reference; Fig. S2), and the resulting total number average molar mass is M_n=41 Kg·mol^{-1}. The SEC curve shows an apparent molecular mass of M_n=36 Kg·mol^{-1} ($Đ$=1.26), and this value seems reasonable in the light of the used PEO calibration. The shift of the molecular mass between the macroinitiator and PBCP is

clearly observable (see Figure S3), which confirms the success of the block polymerization. The yield of the reaction was 0.68 g (33 %).

For the methylation of the PDMAEMA blocks to obtain the quaternized pentablock copolymer *q*PDMAEMA-*b*-PEO-*b*-PPO-*b*-PEO-*b*-*q*PDMAEMA (PBCP), the polymer was dissolved in tetrahydrofuran (THF) and 1.5 molar equivalents (regarding the number of amino groups) of methyl iodide were added under stirring at room temperature. After 15 min, a precipitate developed. The reaction continued for 24 h before it was diluted by water, dialyzed versus aqueous KCl solution (1 mol·L^{-1}) for two days exchanging the solvent three times and another two days versus MilliQ water before the colorless solution was freeze dried over two days. The product was characterized by ^{1}H-NMR spectroscopy in D_2O. Under the assumption of a full conversion to the trimethyl ammonium chloride group, the number average molar mass is expected be at 49.8 Kg·mol^{-1} (Yield: 0.42 g, 60 %).

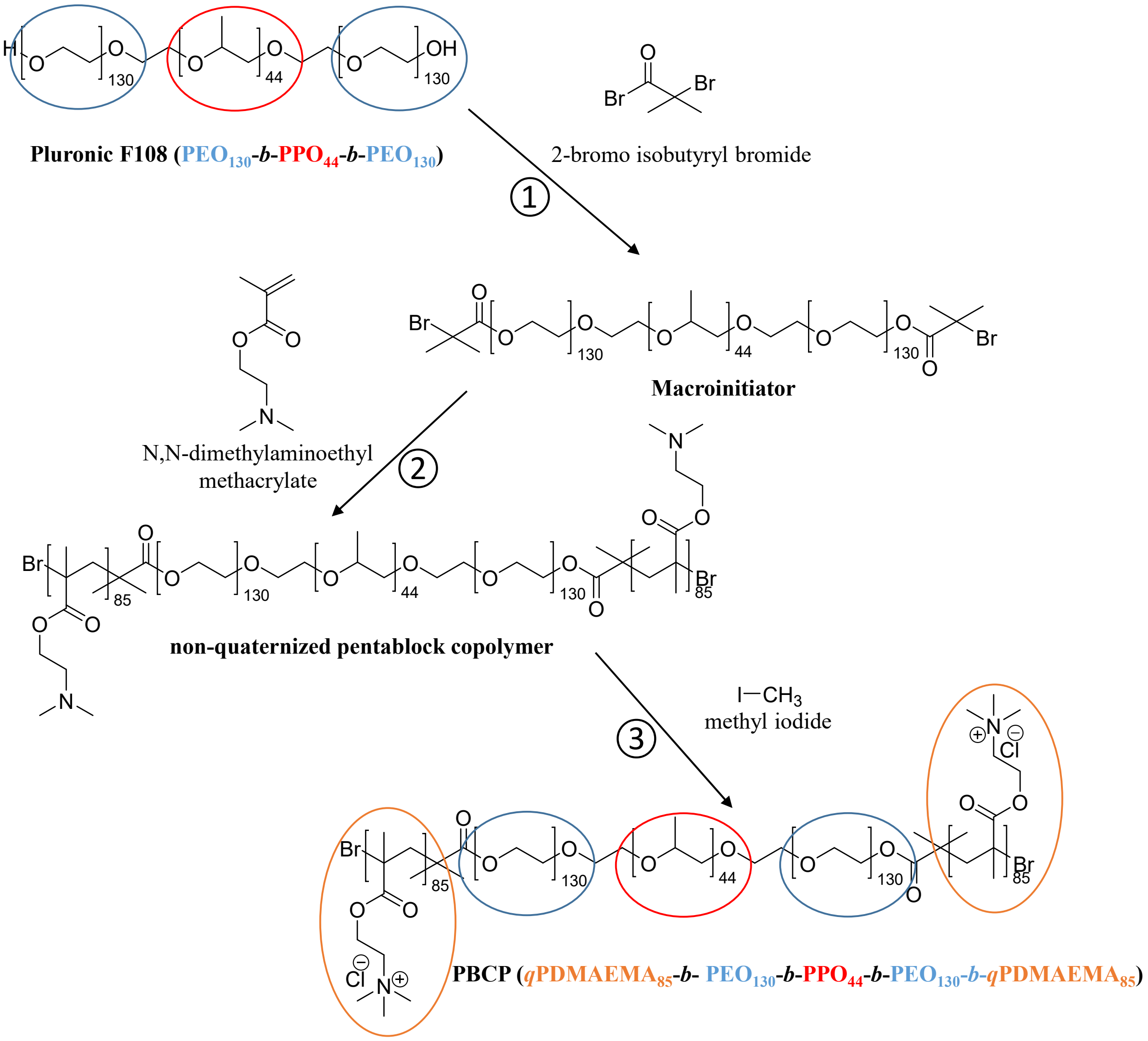

Figure S1: Synthesis of PBCP from the triblock copolymer Pluronic F108 in three steps: ① Esterification to the macroinitiator, ② polymerization to the non-quaternized pentablock copolymer, and ③ quaternization of the PDMAEMA blocks and ion exchange.

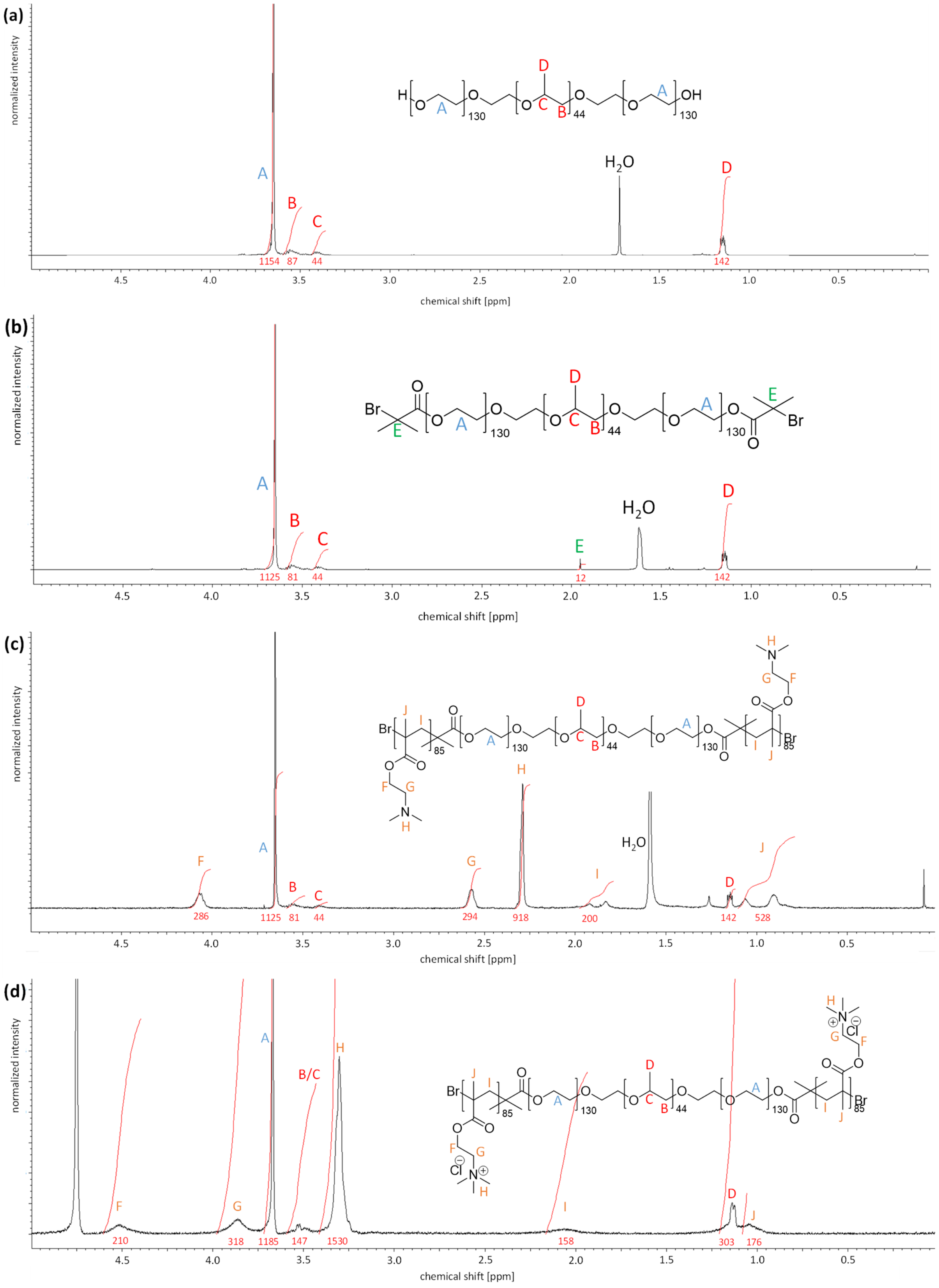

(a)
normalized intensity
A
B
C
D
H2O
1154 87 44
142
4.5 4.0 3.5 3.0 2.5 2.0 1.5 1.0 0.5
chemical shift [ppm]
(b)
normalized intensity
A
B
C
D
E
H2O
1125 81 44
12
142
4.5 4.0 3.5 3.0 2.5 2.0 1.5 1.0 0.5
chemical shift [ppm]
(c)
normalized intensity
F
A
B
C
G
H
I
H2O
D
J
286 1125 81 44 294 918 200 142 528
4.5 4.0 3.5 3.0 2.5 2.0 1.5 1.0 0.5
chemical shift [ppm]
(d)
normalized intensity
A
B/C
H
F
G
I
D
J
210 318 1185 147 1530 158 303 176
4.5 4.0 3.5 3.0 2.5 2.0 1.5 1.0 0.5
chemical shift [ppm]

Figure S2: ^{1}H-NMR-spectra of (a) the triblock copolymer PEO_{130}-*b*-PPO_{44}-*b*-PEO_{130} (Pluronic F108), (b) the macroinitiator after esterification, (c) the non-quaternized pentablock copolymer after the ATRP of DMAEMA, and (d) the quaternized pentablock copolymer (PBCP) after the methylation of the PDMAEMA blocks. (a-c) were recorded in $CDCl_3$, (d) in D_2O as solvent. All products were dialyzed and freeze dried before measurements.

Pluronic F108 (PEO_{130}-*b*-PPO_{44}-*b*-PEO_{130}): δ_H (400 MHz; $CDCl_3$) 3.71-3.61 ($((PEO_{130})_2)$), 3.60-3.48 (PPO_{44} $(CH(CH_3)C\mathit{H}_2)$), 3.45-3.35 (PPO_{44} $(C\mathit{H}(CH_3)CH_2)$, 1.18-1.12 (PPO_{44} $(CH(C\mathit{H}_3)CH_2)$.

Macroinitiator (Br-PEO_{130}-*b*-PPO_{44}-*b*-PEO_{130}-Br): δ_H (400 MHz; $CDCl_3$) 3.71-3.61 ($((PEO_{130})_2)$), 3.60-3.48 (PPO_{44} $(CH(CH_3)C\mathit{H}_2)$), 3.45-3.35 (PPO_{44} $(C\mathit{H}(CH_3)CH_2)$, 1.95 (12H, $(O{=}CC(C\mathit{H}_3)_2Br)_2)$, 1.18-1.12 ($PPO_{44}$ $(CH(C\mathit{H}_3)CH_2)$.

Non-quaternized PBCP ($PDMAEMA_{85}$-*b*-PEO_{130}-*b*-PPO_{44}-*b*-PEO_{130}-*b*-$PDMAEMA_{85}$: δ_H (400 MHz; $CDCl_3$) 4.14-4.00 ($((PDMAEMA_{85})_2$ O-$C\mathit{H}_2)$, 3.71-3.61 ($((PEO_{130})_2)$), 3.58-3.48 (PPO_{44} $(CH(CH_3)C\mathit{H}_2)$), 3.44-3.35 (PPO_{44} $(C\mathit{H}(CH_3)CH_2)$, 2.66-2.52 ($((PDMAEMA_{85})_2$ N-$C\mathit{H}_2)$, 2.35-2.23 ($((PDMAEMA_{85})_2$ N-$(C\mathit{H}_3)_2)$, 1.96-1.76 ($((PDMAEMA_{85})_2$ backbone $C\mathit{H}_2)$, 1.17-1.11 (PPO_{44} $(CH(C\mathit{H}_3)CH_2)$), 1.1-0.85 ($((PDMAEMA_{85})_2$ backbone $C\mathit{H}_3)$.

PBCP (*q*$PDMAEMA_{85}$-*b*-PEO_{130}-*b*-PPO_{44}-*b*-PEO_{130}-b-*q*$PDMAEMA_{85}$): δ_H (400 MHz; D_2O) 4.60-4.35 ((*q*$PDMAEMA_{85})_2$ O-$C\mathit{H}_2$), 3.95-3.75 ((*q*$PDMAEMA_{85})_2$ N-$C\mathit{H}_2$), 3.75-3.60 ($((PEO_{130})_2)$), 3.60-3.45 (PPO_{44} $C\mathit{H}(CH_3)C\mathit{H}_2)$, 3.40-3.20 ((*q*$PDMAEMA_{85})_2$ (N-$C\mathit{H}_3)_3$), 2.20-1.90 ((*q*$PDMAEMA_{85})_2$ backbone $C\mathit{H}_2$), 1.25-0.90 (*q*$PDMAEMA_{85}$ backbone $C\mathit{H}_3$/ PPO_{44} $CH(C\mathit{H}_3)CH_2)$.

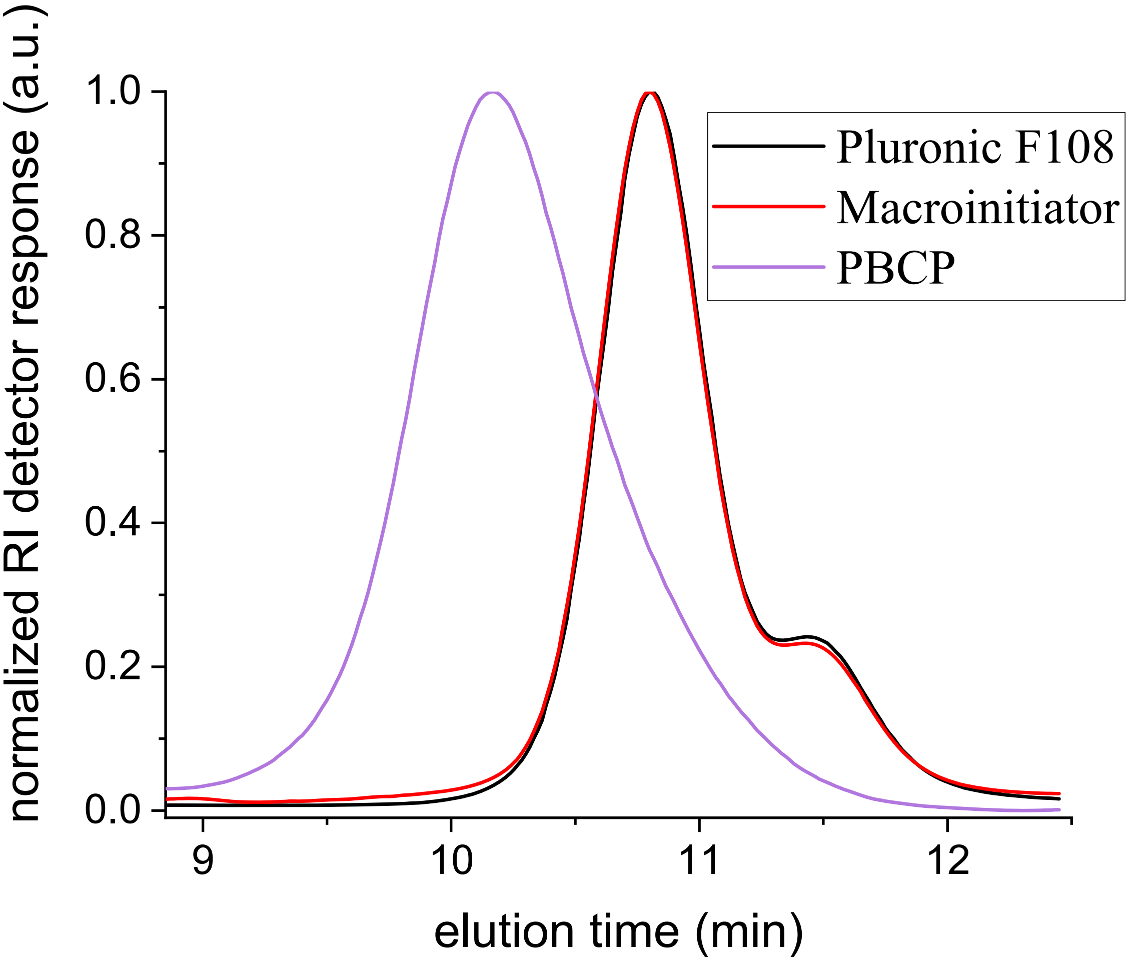

Figure S3: Size exclusion chromatogram of the triblock copolymers PEO_{130}-*b*-PPO_{44}-*b*-PEO_{130} (Pluronic F108), the macroinitiator after esterification, and the non-quaternized pentablock copolymer *q*$PDMAEMA_{85}$-*b*-PEO_{130}-*b*-PPO_{44}-*b*-PEO_{130}-*b*-*q*$PDMAEMA_{85}$. DMAc was used as solvent.

Dynamic Light Scattering - Averaging time-dependent hydrodynamic radii:

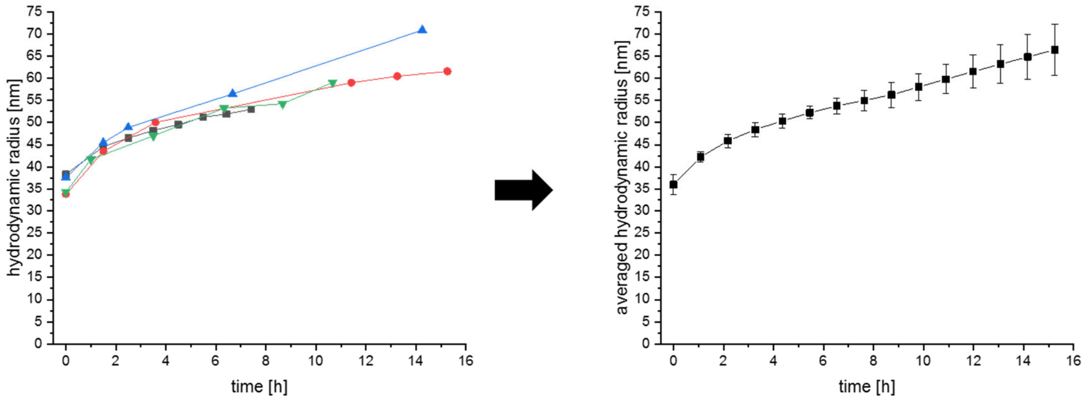

Figure S4: Averaging of the time-dependent hydrodynamic radii. (left) 4 measurements of the hydrodynamic radius development depending on the time the samples were hold at 27.5 °C. All sample solutions were taken from a stock solution prepared below 15 °C and stored at 4 °C. (right) averaged development of the hydrodynamic radius. The curves were linearly interpolated between the measuring points and 12 equidistant points were calculated including error values. The averaging was performed by the software OriginPro 2018b.

Structural Elucidation – Height Profiles as Additional AFM-images:

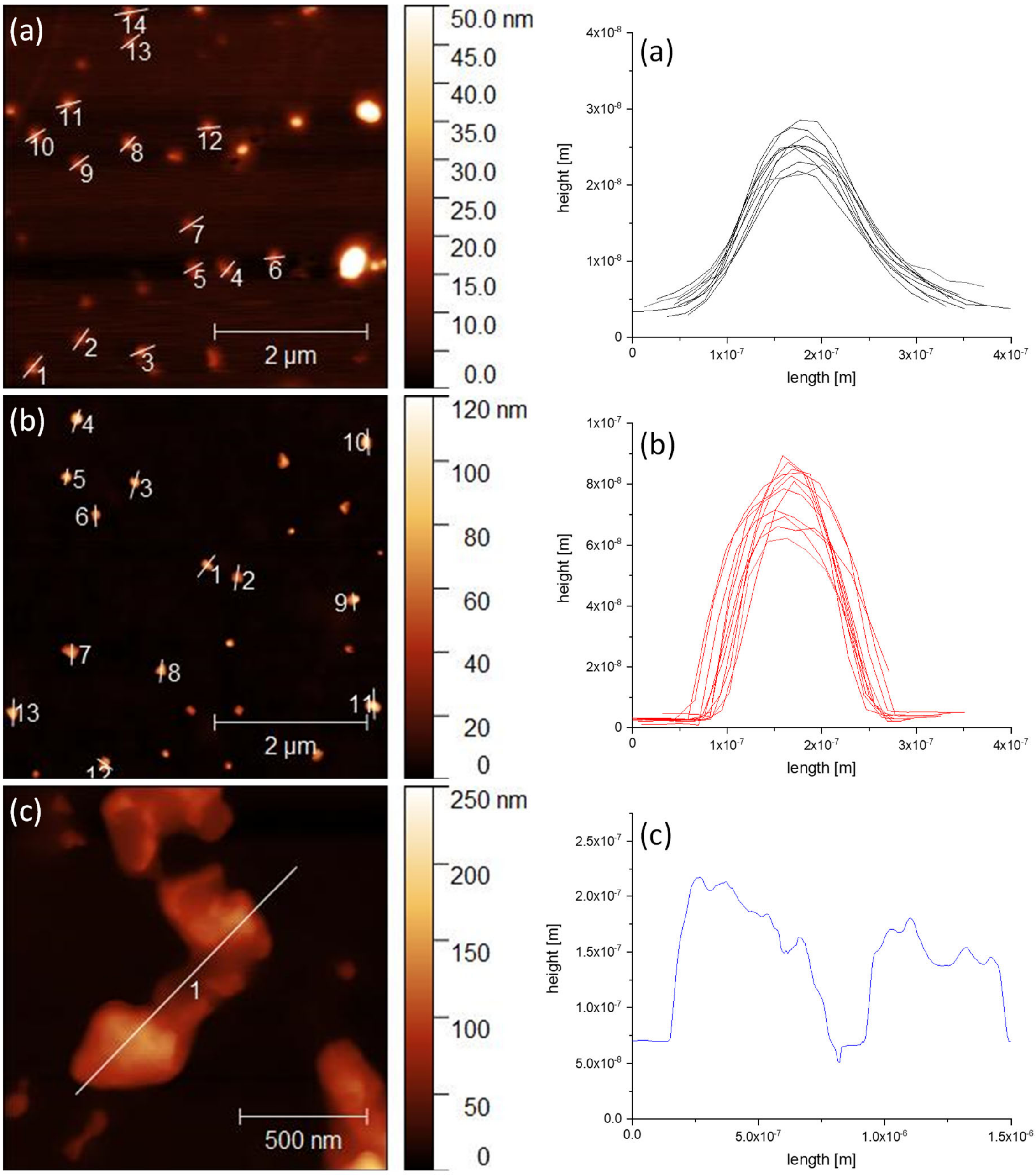

Figure S5: AFM images including height profiles of an aqueous solution of PBCP (1 g·L^{-1}), $K_3[Fe(CN)_6]$ (0.0025 mol·L^{-1}), and KCl (0.1 mol·L^{-1}), prepared at temperatures below 15 °C and transferred to a silicon wafer after (a) 0 h, (b) 7 h, and (c) 24 h at 27.5 °C.

Structural Elucidation - Small angle X-ray scattering: Small Angle X-Ray Scattering (SAXS) data was recorded to trace the solution behavior before and after the increase of temperature.[3] Hence, small angle X-ray scattering curves of a PBCP/ $K_3[Fe(CN)_6]$/ KCl solution at 25 °C and at 35 °C (below and above the threshold temperature of 27 °C) are depicted in Figure 5. The scattering intensity I(q) was fitted by different models of the type

$$I(\mathrm{q}) = P(\mathrm{q}) \cdot S(\mathrm{q}) + \mathrm{background}, \qquad \text{Equation S 1}$$

where P(q) (= $F(\mathrm{q})^2$) is the form factor and S(q) is the structure factor.

At 25 °C, a form factor for core-shell particles[4] (see Equation S 2) was used under the assumption of flower-like micelles consisting of a qPDMAEMA/ferricyanide core and a PEO/PPO shell.

$$\sqrt{\mathrm{P(q)}} = \mathrm{F(q)} = \frac{3}{\mathrm{V_S}} \cdot \left[\mathrm{V_C}(\rho_\mathrm{C} - \rho_\mathrm{S}) \frac{\sin(\mathrm{qr_C}) - \mathrm{qr_C} \cdot \cos(\mathrm{qr_C})}{(\mathrm{qr_C})^3} + \mathrm{V_S}(\rho_\mathrm{S} - \rho_\mathrm{solv}) \frac{\sin(\mathrm{qr_S}) - \mathrm{qr_S} \cdot \cos(\mathrm{qr_S})}{(\mathrm{qr_S})^3}\right], \qquad \text{Equation S 2}$$

where V_S is the volume of the whole particle, V_C the volume of the core, r_S the radius of the particle, r_C the radius of the core, and ρ_C, ρ_S, and ρ_{solv} the scattering length densities of the core, the shell and the solvent.

Additionally, a sticky-hard-sphere structure factor[5] was used for the interaction of the PPO blocks. The fit parameters are listed in Table S1. Since scattering length densities of core and shell are not known, they were initially fitted together with the scale factor and the background and fixed at the obtained values ($\rho_C = 14.121 \cdot 10^{-6}$ nm^{-2}, $\rho_S = 9.59 \cdot 10^{-6}$ nm^{-2}, fixed $\rho_{solv} = 9.452 \cdot 10^{-6}$ nm^{-2}).

Table S 1: Fit parameters of the SAXS curves:

25°C (core-shell form factor with sticky-hard-sphere structure factor)

**core radius (nm)**	10.43	± 0.13
**shell thickness (nm)**	15.91	± 0.77
**radius distribution (Gaussian)**	0.12	-
**shell thickness distribution**	0.00	-
**radius$_{effective}$ (nm)**	26.34	-
**volume fraction**	0.07	± 0.01
**perturbation**	0.1	± 25.8
**stickiness**	539.57	± 1e+08

35°C (hard sphere form factor, i.e. shell thickness set to 0, and hard sphere structure factor)

**core radius (nm)**	13.03	± 0.04
**radius distribution (Gaussian)**	1	± 0.007
**radius$_{effective}$ (nm)**	16.88	-
**volume fraction**	0.293	± 0

Since scattering length densities of core and shell are not known, the values of the flower fit above were used also for the bouquet fit. It is to mention that scattering data are distorted by X-ray fluorescence at the used wavelength. Hence, the evaluation shows a tendency of the temperature dependent structural development, but are not assumed to be absolutely quantitatively accurate.

Alternative Mechanism of Irreversible Coagulation: It is known that non-quaternized PDMAEMA and PPO in spatial vicinity (neighboring arms in miktoarm stars) can form complexes.[6,7] This possible complexation might contribute to the noteworthy solution behavior of the pentablock copolymers. As a possible scenario, the originally spherical (flower-like) micelles

could generate precipitating networks of worm-like micelles upon backfolding and complexation of the PPO block with the *q*PDMAEMA core, which would increase the hydrophobic domain being suitable for larger morphology changes. Even under the assumption that *q*PDMAEMA in presence of ferricyanide ions shows a similar complexation behavior toward PPO, the fraction of complexed PPO should be reduced because of the length of the PEO spacer block between PPO and *q*PDMAEMA, which separates the two blocks making a complexation less favorable.[8] The plausibility of this mechanism was checked by ^{1}H-NMR spectroscopy in D_2O of the PBCP solution with reduced amounts of potassium ferricyanide (0.25 charge equivalents considering the ammonium groups; Fig. S6). Because of the paramagnetism of ferricyanides, the resolution of the spectra is significantly reduced in contrast to the pure spectra without ferricyanide. The fact that the PPO (at 1.2 ppm) and PEO (at 3.7 ppm) peaks are least reduced indicates a spatial distance to the ferricyanide ions located in the immobilized core, which confirms the flower-like structure of the micelles. In contrast, signals of the *q*PDMAEMA blocks vanish even at small amounts of ferricyanide. The integral ratio between the PEO protons (at 3.7 ppm) and the most prominent PPO protons (at 1.2 ppm) changes only slightly from 8 to 7 after heating, Since hardly any reduction of the intensity of the PPO signal (compared to the PEO signals) was detected after increasing the temperature and cooling back to room temperature, consequently inducing a precipitation of the system, the PPO/*q*PDMAEMA complexation mechanism seems unlikely to explain the special thermoresponsive behavior of the investigated system. Additionally, the comparison with the water signal (at 4.7 ppm) points to only minor changes of the "NMR-availability" of both PEO and PPO protons before and after heating, despite the resulting limited colloidal stability reflected in a further line broadening. Hence, thermo-induced complexation between PPO and qPDMAEMA does not seem to be a major reason for the irreversible transition.

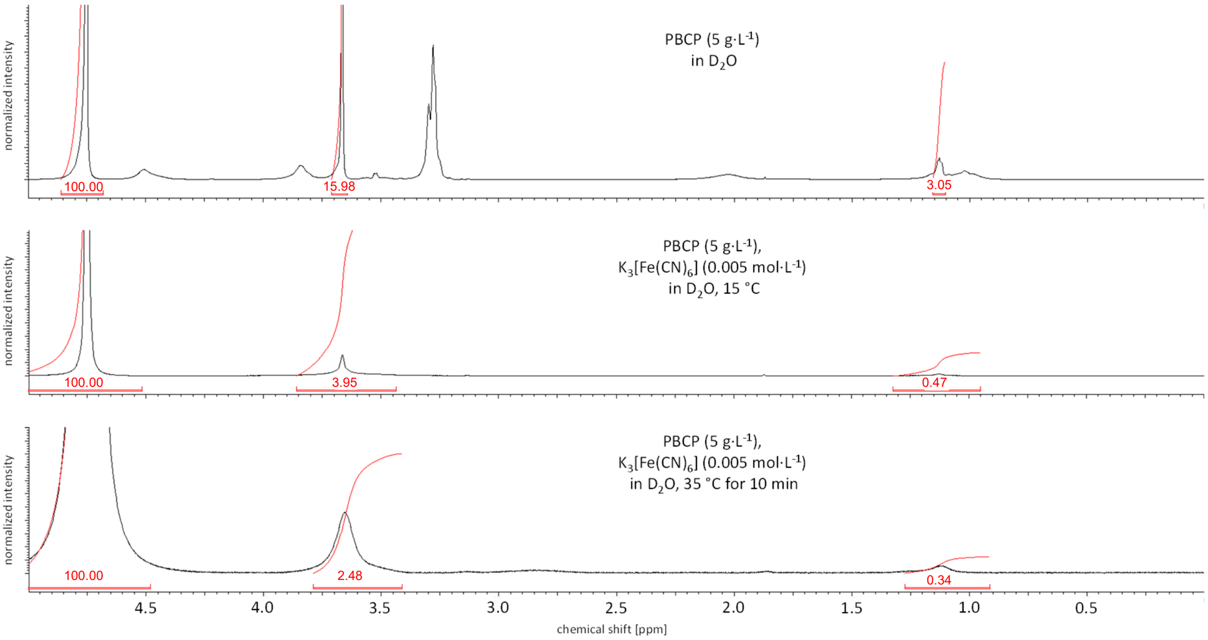

Figure S6: ^{1}H-NMR spectra of the PBCP (5 g·L^{-1}) in D_2O with no additives (above), with ferricyanide (0.005 mol·L^{-1}) prepared below 15 °C, measured both at 15 °C, once before and once after heating the sample to 35 °C for 10 min.

.

Formation of Prussian Blue: In previously discussed experiments, ferricyanide ions were added in excess to assure the hydrophobicity of the polycationic blocks. To reduce the formation of PB crystals in the continuous phase outside of the micellar cores, the concentration of ferricyanide ions was reduced to a half equivalent with respect to the charge of *q*PDMAEMA. The solution turned blue because of the addition of iron(II) ions and the consequent formation of PB. A blue precipitate formed within one night at the bottom of the sample leaving a transparent blue upper phase. The precipitate likely consists of PB crystals formed with a remnant of molecular dispersed ferricyanide ions. Hence, there is a ferricyanide competition between its interaction with the

polymer and the ferrous ions. Apparently, a part of polymer is still linked to the inorganic PB material, providing a blue/transparent supernatant.

References

(1) Steinschulte, A. A.; Xu, W.; Draber, F.; Hebbeker, P.; Jung, A.; Bogdanovski, D.; Schneider, S.; Tsukruk, V. V.; Plamper, F. A. Interface-Enforced Complexation Between Copolymer Blocks. *Soft Matter* **2015**, *11* (18), 3559–3565

(2) Bütün, V.; Armes, S. P.; Billingham, N. C. Selective Quaternization of 2-(Dimethylamino)ethyl Methacrylate Residues in Tertiary Amine Methacrylate Diblock Copolymers. *Macromolecules* **2001**, *34* (5), 1148–1159.

(3) *Software SasView*: *http://www.sasview.org/* (accessed 15th of August 2023).

(4) Guinier, A.; Fournet, G. *Small-angle scattering of X-rays*; John Wiley and Sons, 1955. 1-276

(5) Menon, S. V. G.; Manohar, C.; Rao, K. S. A New Interpretation of the Sticky Hard Sphere Model. *Journal of Chemical Physics* **1991**, *95* (12), 9186–9190.

(6) Steinschulte, A. A.; Schulte, B.; Erberich, M.; Borisov, O. V.; Plamper, F. A. Unimolecular Janus Micelles by Microenvironment-Induced, Internal Complexation. *ACS Macro Letters* **2012**, *1* (4), 504–507.

(7) Hebbeker, P.; Steinschulte, A. A.; Schneider, S.; Okuda, J.; Möller, M.; Plamper, F. A.; Schneider, S. Complexation in Weakly Attractive Copolymers with Varying Composition and Topology:Linking Fluorescence Experiments and Molecular Monte Carlo Simulations. *Macromolecules* **2016**, *49* (22), 8748–8757.

(8) Hebbeker, P.; Langen, T. G.; Plamper, F. A.; Schneider, S. Spacer Chains Prevent the Intramolecular Complexation in Miktoarm Star Polymers. *The Journal of Physical Chemistry B* **2018**, *122* (17), 4729–4736.
